# Midwifery and General Practice

**Published:** 1899-04-08

**Authors:** 


### Editor's Letter-Box.

[Our Correspondents are reminded that prolixity is a great bar to publication, and that brevity of style and conciseness of statement greatly facilitate early insertion.]

### MIDWIFERY AND GENERAL PRACTICE.

Miss Rosalind Paget writes:—Will you kindly note *re* "Midwifery and General Practice," in The Hospital of the 25th ult., that "The Midwives' Society" is *of Manchester*. If this is not mentioned people are certain to consider that you are alluding to the Midwives' Institution. These societies have no connection with each other, and the Midwives' Society of Manchester is opposing the present Bill. *We* are not, as we think almost any legislation better than none.

